# Triple‐Coupling Regulation Enables Dendrite‐Free Subzero Aqueous Zn‐Ion Batteries

**DOI:** 10.1002/advs.78147

**Published:** 2026-10-08

**Authors:** Mengyang Dong, Zhibao Yang, Lina Pan, Jie Qu, Shangshu Qian, Jessica Hamilton, Bernt Johannessen, Yuhui Tian, Chao Xing

**Affiliations:** ^1^ College of Chemistry and Chemical Engineering Hunan Normal University Changsha China; ^2^ UQ Dow Centre for Sustainable Engineering Innovation School of Chemical Engineering The University of Queensland St Lucia Queensland Australia; ^3^ College of Chemical and Biological Engineering Zhejiang University Hangzhou China; ^4^ Australian Synchrotron ANSTO Clayton VIC Australia; ^5^ School of Molecular and Life Sciences Curtin University Bentley Western Australia Australia

**Keywords:** aqueous zinc‐ion batteries, crystallographic orientation, deep eutectic electrolytes, interfacial chemistry, subzero‐temperature energy storage, Zn^2+^ solvation structure

## Abstract

Aqueous Zn‐ion batteries (AZIBs) suffer severe degradation at subzero temperatures due to electrolyte freezing, sluggish Zn^2+^ kinetics, and dendrite‐induced interfacial instability. Herein, a triple‐coupling regulation strategy integrating solvation structure, interfacial chemistry, and crystallographic orientation through a formamide‐ethanol deep eutectic electrolyte (ZEFW2) was developed. Molecular dynamics simulations and spectroscopic analyses reveal that ZEFW2 redistributes the primary Zn^2+^ solvation environment, reducing H_2_O and acetate coordination while introducing formamide coordination and maintaining favorable Zn^2+^ transport. Depth‐resolved XPS further reveals a chemically reconstructed Zn interphase containing Zn‐O‐related inorganic species and persistent N‐ and C‐containing electrolyte‐derived species. The modified solvation and interfacial environments regulate Zn nucleation and promote preferential Zn deposition along the (101) facet, enabling more homogeneous Zn growth and suppressing dendritic morphology. Consequently, Zn||Zn symmetric cells achieve 1400 h of cycling at −20°C under 1 mA cm^−2^ and 10 mAh cm^−2^, with a Coulombic efficiency of 99.78% over 1200 cycles. Zn||NVO full cells retain 74% of their initial capacity after 1600 cycles at −20°C, while pouch cells demonstrate stable operation at both room and subzero temperatures. This work establishes a molecular‐to‐interfacial triple‐coupling strategy for temperature‐resilient AZIBs.

## Graphical abstract

A formamide‐ethanol deep eutectic electrolyte enables triple‐coupling regulation of solvation structure, interfacial chemistry, and crystallographic Zn deposition. The reconstructed Zn^2+^ solvation reduces water activity and suppresses side reactions, while a robust hybrid SEI stabilizes the interface. This coupling directs (101)‐facet Zn growth, achieving dendrite‐free cycling and long‐term stability of aqueous Zn‐ion batteries at ‐20°C.

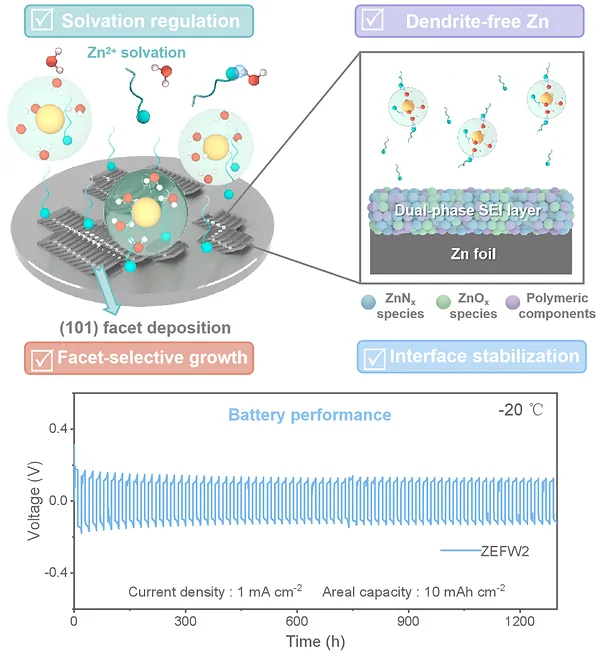

## 1 Introduction

Rechargeable aqueous zinc‐ion batteries (AZIBs) have emerged as promising candidates for next‐generation stationary energy storage systems, owing to the abundance of Zn resources, environmental benignity, low redox potential (−0.762 V vs. standard hydrogen electrode (SHE)), high theoretical capacity (820 mAh g^−1^), and high theoretical volumetric capacity (5855 mAh cm^−3^). These merits make AZIBs a cost‐effective alternative to lithium‐based chemistry for large‐scale energy storage applications [1, 2]. However, the practical implementation of AZIBs is severely constrained by intrinsic challenges, particularly at subzero temperatures (<0°C). The Zn anode exhibits inherent instability during repeated plating and stripping cycles, manifested in uncontrolled dendritic growth, parasitic hydrogen evolution reaction (HER), and progressive interfacial degradation [3, 4]. Concurrently, conventional aqueous electrolytes suffer from increased viscosity and crystallization of free water at low temperatures, which induce interfacial pH fluctuations, local corrosion, and accumulation of insulating by‐products (e.g., Zn_4_(OH)_6_(CO_3_)·H_2_O) [5, 6, 7]. These drawbacks are mutually reinforcing under subzero conditions: sluggish Zn^2+^ desolvation kinetics, elevated ionic resistance, electrolyte freezing, and loss of ionic conductivity conjointly accelerate dendrite formation, deteriorate Zn reversibility, increase polarization, and ultimately reduce coulombic efficiency (CE), rendering stable operation extremely difficult [8, 9, 10].

High‐concentration “water‐in‐salt” electrolytes and fluorinated additives have been explored to alleviate these issues [11, 12]. Although these approaches can suppress freezing and interfacial side reactions to some extent, their reliance on expensive salts or fluorinated components compromises sustainability and limits their feasibility for large‐scale deployment. Consequently, attention has increasingly shifted toward regulating the solvation chemistry of Zn^2+^ itself, which offers a more intrinsic route to simultaneously addressing electrolyte crystallization and parasitic hydrogen evolution at subzero temperatures [13, 14, 15]. From a coordination‐chemistry perspective, Zn^2+^ in conventional aqueous electrolytes behaves as a strongly hydrated Lewis acid, predominantly existing as an octahedral [Zn(H_2_O)_6_]^2+^ complex. The high stability of this hydration sheath imposes a large energetic barrier for interfacial desolvation, which slows charge‐transfer kinetics and becomes increasingly restrictive at subzero temperatures as the hydrogen‐bond network of water loses mobility [16, 17]. The resulting rigidity elevates ion‐transport resistance, induces heterogeneous Zn nucleation, and accelerates dendrite formation. At the same time, strong Zn‐O coordination polarizes bound water molecules, increasing local water activity and facilitating proton‐coupled side reactions as well as the formation of ordered clusters that precede electrolyte freezing. These coupled phenomena reveal that the low‐temperature failure of AZIBs is fundamentally rooted in the nature of the Zn^2+^ solvation sheath, making its deliberate reconstruction a critical prerequisite for stabilizing interfacial processes [18, 19, 20, 21, 22].

Beyond solvation regulation, stabilizing the Zn metal anode under subzero conditions requires simultaneous control over interfacial chemistry and crystallographic deposition behavior. Constructing a compact, ion‐conductive solid electrolyte interphase (SEI) has been widely recognized as an effective strategy to suppress parasitic reactions and dendritic growth [23, 24]. Meanwhile, increasing evidence indicates that Zn deposition is intrinsically anisotropic and strongly facet‐dependent [25, 26, 27]. In particular, preferential growth along the thermodynamically favorable (101) crystallographic facet can mitigate dendrite formation due to its high surface energy, isotropic growth pathway, and dense atomic packing [25, 28]. Importantly, Zn^2+^ solvation structure, interfacial SEI chemistry, and crystallographic orientation are not independent; rather, they are strongly coupled through coordination chemistry and interfacial energetics. This interdependence highlights the necessity of a unified regulation framework capable of simultaneously addressing solvation dynamics, interface stability, and deposition pathways, especially under kinetically constrained subzero conditions. These considerations motivate a “triple‐coupling regulation” strategy that unifies solvation reconstruction, interfacial‐chemistry engineering, and facet‐selective crystallographic regulation to stabilize Zn metal anodes. However, realizing such integrated regulation remains challenging and calls for electrolyte platforms that can simultaneously manipulate hydrogen‐bond networks, coordination environments, and surface energetics in a controllable manner [10, 29, 30].

Deep eutectic electrolytes (DEEs) are well suited to satisfy these requirements. Composed of hydrogen bond donors and acceptors with strong Lewis acidity/basicity, DEEs enable extensive tunability of solvation structure and hydrogen‐bonding dynamics [31, 32]. Their intrinsic ability to reduce water activity [33], regulate Zn^2+^ coordination environments [23, 34], and promote in situ formation of inorganic‐organic hybrid interphases [35] provides a natural pathway to couple solvation reconstruction with SEI formation. Moreover, the strong and selective adsorption of eutectic components on metal surfaces can modulate surface energies of different crystallographic facets, offering a feasible route to facet‐directed Zn nucleation and growth [36, 37, 38]. Incorporating a controlled amount of water further mitigates the high viscosity typically associated with DEEs, ensuring sufficient ionic conductivity and favorable kinetics even at subzero temperatures [39].

Despite recent progress, most low‐temperature aqueous Zn‐ion battery studies rely on single‐factor regulation, such as solvation modification or SEI engineering in isolation, thus failing to simultaneously mitigate electrolyte freezing, sluggish Zn^2+^ kinetics, and dendrite‐induced interfacial instability. This lack of integrated regulation fundamentally limits the performance of subzero AZIBs. To bridge this gap, a formamide‐ethanol‐zinc acetate deep eutectic electrolyte (ZEFW2) is developed to realize a triple‐coupling regulation strategy that coherently links Zn^2+^ solvation structure, interfacial chemistry, and crystallographic deposition behavior. Synchrotron X‐ray absorption spectroscopy (XAS), Raman/FTIR spectroscopy, ^2^H NMR, and density functional theory (DFT) calculations comprehensively reveal that Zn‐N coordination reconstructs the Zn^2+^ solvation sheath, induces a robust hybrid SEI, and directs preferential (101) facet Zn deposition. As a result, ZEFW2 enables ultra‐stable Zn cycling at ‐20°C with a lifespan of 1400 h at 1 mA cm^−2^ and 10 mAh cm^−2^, outperforming recently reported subzero AZIB electrolytes. More importantly, the clarified mechanistic pathway, spanning solvation reconstruction, interfacial stabilization, and facet‐selective deposition, establishes a general design principle for temperature‐resilient metal‐ion batteries.

## 2 Results and Discussion

### 2.1 Solvation Engineering of Zn^2+^

As schematically illustrated in Figure 1, the triple‐coupling regulation strategy starts from solvation structure reconstruction, which subsequently governs interfacial chemistry and crystallographic Zn deposition behavior. The structural evolution is conceptually depicted in Figure 1a,b, while the quantitative Zn─Zn distances discussed here are derived from synchrotron X‐ray absorption spectroscopy (EXAFS) fitting presented in later sections. Specifically, conventional electrolytes exhibit a shorter Zn─Zn distance (∼2.18 Å), indicative of dense metallic aggregation that favors anisotropic nucleation, whereas the ZEFW2 electrolyte shows an expanded Zn‐Zn distance (∼2.37 Å), suggesting a more spatially relaxed and partially coordinated Zn environment that promotes uniform deposition. The solvation structure is the core of triple‐coupling regulation, as it directly governs interfacial chemistry and Zn deposition behavior. In conventional aqueous electrolytes, Zn^2+^ is typically coordinated by six water molecules in an octahedral solvation structure ([Zn(H_2_O)_6_]^2+^), leading to intrinsic drawbacks of free water crystallization at subzero temperatures and enhanced hydrogen evolution reactions (HER). To overcome these limitations, we designed an ethanol‐formamide (EF) deep eutectic system in which one water molecule in the primary solvation sheath is substituted by formamide (Figure 1d), forming a Zn‐N coordinated solvation structure that fundamentally modulates the hydrogen‐bond network and suppresses free water activity.

**FIGURE 1:**
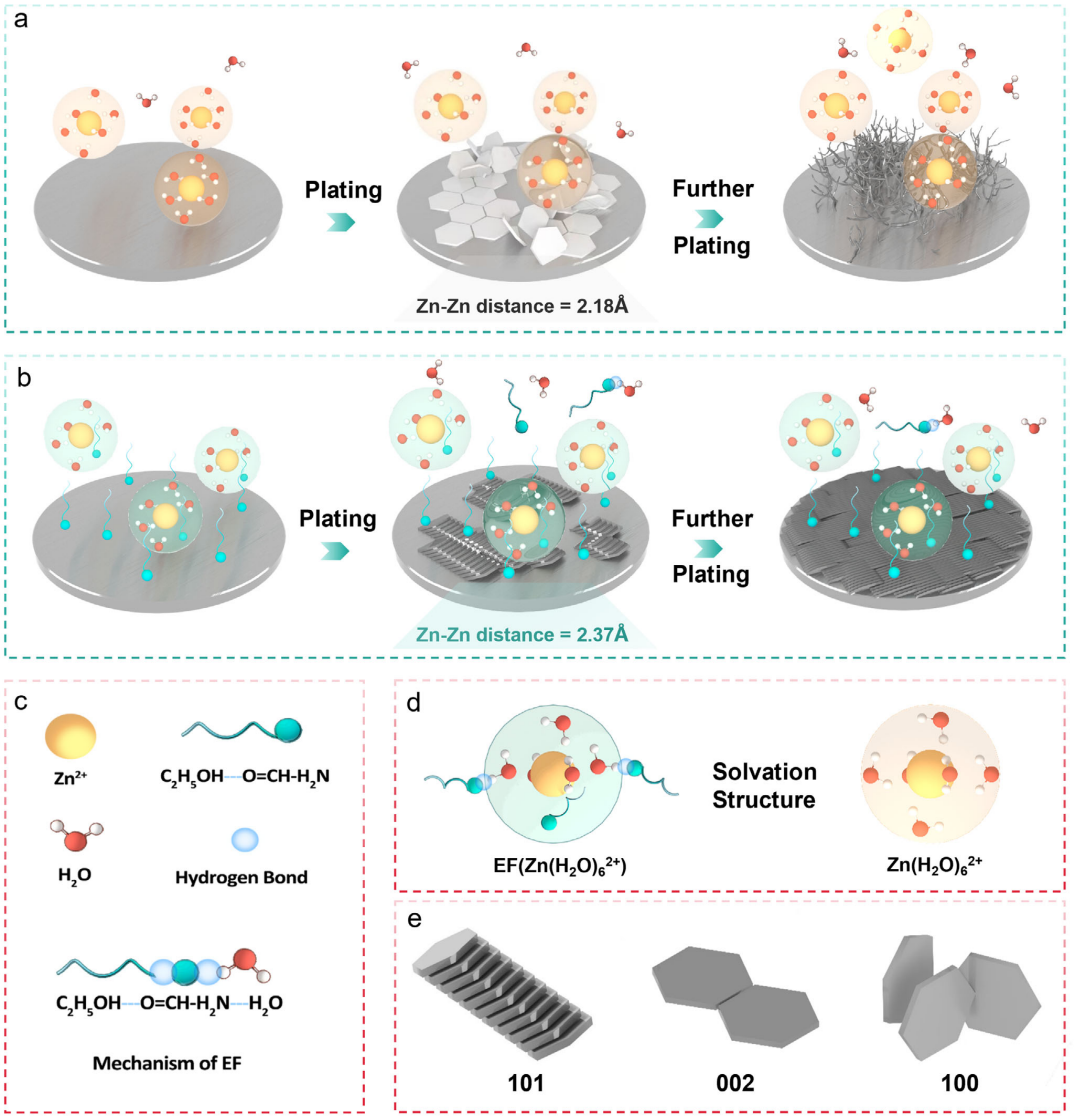
Mechanism schema of regulating Zn deposition additives by (a) Zn(OAc)_2_ and (b) ZEFW2. (c) and (d) Schematic diagram of different molecular structures. (e) Schematic diagram of different crystal planes.

Differential scanning calorimetry (DSC) measurements (Figure 2a) revealed a pronounced endothermic peak between 0°C and 5°C for the Zn(OAc)_2_ aqueous electrolyte, corresponding to the freezing of bulk water. In sharp contrast, all EF‐based deep eutectic formulations, including ZEFW2, exhibited no discernible thermal transitions over the −75°C to 25°C range, demonstrating a broad thermal stability window and excellent resistance to freezing [40]. This finding was further corroborated by optical observations (Figure S1): while Zn(OAc)_2_ showed partial solidification even at 5°C, all deep eutectic electrolytes remained fully liquid after standing for 1 h at −40°C. The superior liquid‐state retention arises from a synergistic hydrogen‐bond network among FA, EtOH, and residual water, which disrupts the tetrahedral ordering of H_2_O molecules and effectively suppresses ice nucleation at low temperature. It should be noted that the absence of a thermal transition over the DSC range does not by itself establish electrochemical operation across the same temperature window; therefore, the electrochemical performance of ZEFW2 was conservatively evaluated down to −20°C in this work. Given the introduction of organic components, the flammability of ZEFW2 was also evaluated. Flame tests (Figure S2a,b) demonstrated that a glass fiber separator soaked in ZEFW2 could not be ignited, whereas an ethanol‐rich EF formulation burned readily. The non‐flammability of ZEFW2 is attributed to its relatively high‐water content and robust H‐bonding interactions, which lower the vapor pressure and inhibit the release of flammable vapors. These characteristics collectively highlight the favorable low‐temperature thermal behavior and safety characteristics of ZEFW2, while its electrochemical performance is discussed within the experimentally validated temperature range below. Thermogravimetric analysis (TGA, Figure S3) further revealed suppressed low‐temperature mass loss and a broader, higher‐temperature volatilization regime for ZEFW2 compared with Zn(OAc)_2_, with ZEFW2 retaining 60.4% of its initial mass at 96°C, whereas Zn(OAc)_2_ retained only 19.0% at 115°C. This behavior is consistent with the strengthened FA/EtOH/H_2_O hydrogen‐bond network and Zn^2+^‐solvent interactions, which reduce readily volatilizable species and shift their release toward higher temperatures. To elucidate the molecular interactions underlying the deep eutectic electrolyte design, we first examined the hydrogen‐bonding between formamide (FA) and ethanol (EtOH) using FTIR spectroscopy (Figure 2b). Pure FA exhibits a C═O stretching vibration at 1662 cm^−1^, which progressively shifts to higher wavenumbers (1671 cm^−1^) upon EtOH addition. Such concurrent shifts in both C═O vibrations clearly indicate the formation of strong intermolecular hydrogen bonds, primarily through C═O···OH interactions [41]. These interactions reorganize the hydrogen‐bond network, providing the structural basis for the eutectic mixture and enabling its unique thermophysical properties.

**FIGURE 2:**
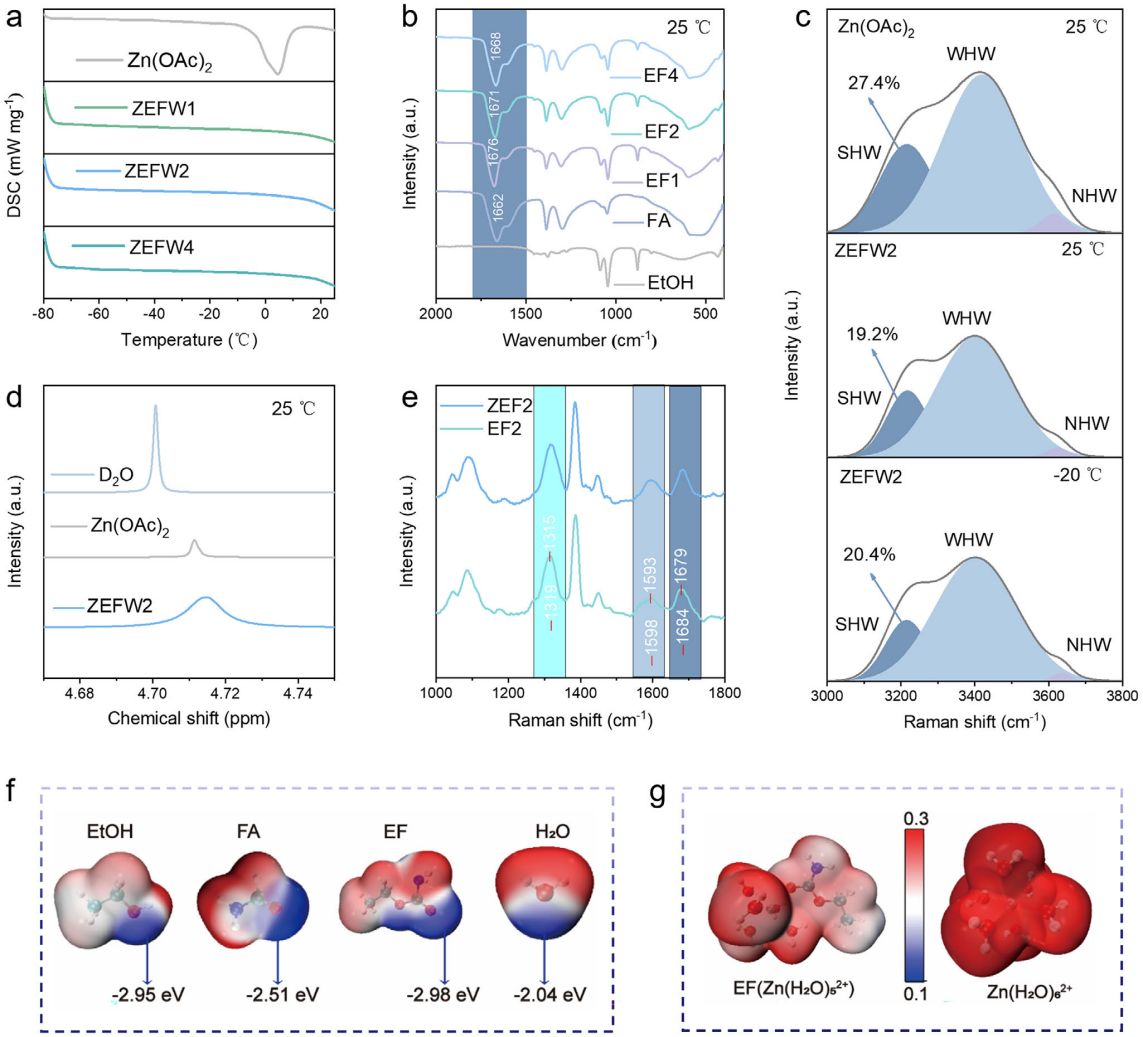
(a) DSC curves of different electrolytes. (b) FTIR spectra for pure EtOH, pure FA and the mixture with different molar ratios between EtOH and FA at 25°C. (c) The fitted O─H stretching vibration in the Raman spectra for different electrolytes at different temperatures. (d) ^2^H NMR spectra of different electrolytes at 25°C. (e) Raman spectra for ZEFW2 and EFW2 at 25°C. (f) Electrostatic potential diagrams of EtOH, FA, EF and H_2_O. (g) Electrostatic potential diagrams of EF(Zn(H_2_O)_5_^2+^) and Zn(H_2_O)_6_^2+^.

Building on this understanding, Raman spectroscopy was employed to prove how the introduction of EF alters the hydrogen‐bond network in aqueous media. Deconvolution of the O‐H stretching region into strongly hydrogen‐bonded water (SHW), weakly hydrogen‐bonded water (WHW), and non‐hydrogen‐bonded water (NHW) (Figure 2c and Table S1) reveals that ZEFW2 contains a markedly lower SHW fraction (19.2%) than the Zn(OAc)_2_ electrolyte (27.4%), signifying reduced free water content and suppressed parasitic reactions [42]. Moreover, the SHW fraction in ZEFW2 remains essentially unchanged from 25°C to −20°C, suggesting that the hydrogen‐bond network is highly resilient to temperature fluctuations [43]. To further probe solvation dynamics, ^2^H NMR measurements were conducted at 25°C (Figure 2d). The D_2_O resonance at 4.701 ppm shifts downfield in Zn(OAc)_2_ electrolyte due to coordination‐induced de‐shielding by Zn^2+^, and this shift becomes even more pronounced in ZEFW2 (4.73 ppm). Such an enhanced shift indicates that stronger FA/EtOH‐H_2_O hydrogen bonding reduces direct Zn^2+^‐H_2_O coordination, thereby reshaping the primary solvation sheath [44]. Raman analysis further supports this conclusion: upon adding Zn(OAc)_2_ to EFW2 (Figure 2e), the C═O and ─CN stretching bands undergo distinct blue shifts (from 1671 to 1678 cm^−1^ for C═O, from 1495 to 1502 cm^−1^ for ─CN), confirming that EF molecules preferentially coordinate with Zn^2+^. These experimental observations are reinforced by density functional theory (DFT) calculations (Figure 2f,g). Electrostatic potential mapping shows that EF possesses the most negative local potential (−2.98 eV) among all solvent components, exceeding that of water (−2.04 eV). Furthermore, EF‐Zn^2+^ complexes exhibit a reduced surface potential compared to Zn(H_2_O)_6_^2+^, indicating a thermodynamically favorable substitution of water by EF in the primary solvation shell, which is expected to facilitate Zn^2+^ migration. Finally, the macroscopic electrochemical response validates these molecular‐level insights. Electrochemical impedance spectroscopy using stainless steel||stainless steel symmetric cells further corroborated the favorable transport characteristics of ZEFW2, which exhibited a relatively low impedance at both temperatures, particularly at −20°C (Figure S4). At both 25°C and −20°C, ZEFW2 achieves the highest ionic conductivity (12.8 mS cm^−1^ at 25°C, 3.2 mS cm^−1^ at −20°C) and Zn^2+^ transference number (0.433 at 25°C, 0.41 at −20°C) among tested electrolytes (Figures S5–S7). This proves that the optimized solvation environment could enhance low‐temperature ion transport and alleviate the interfacial concentration gradient [45].

### 2.2 Solvation‐to‐Interfacial Chemical Coupling

The molecular‐level reconstruction of the electrolyte environment was further investigated by all‐atom molecular dynamics (MD) simulations. Representative configurations after equilibration show a distinct difference in the local coordination environment of Zn^2+^ between the conventional Zn(OAc)_2_ and ZEFW2 electrolytes (Figure 3a). In Zn(OAc)_2_ Zn^2+^ is predominantly surrounded by H_2_O and OAc^−^, whereas the introduction of formamide (FA) and ethanol (EtOH) in ZEFW2 produces a more diverse local solvation environment. Such redistribution of solvent and anion coordination is consistent with the established role of electrolyte solvation chemistry in controlling Zn^2+^ transport, desolvation, and interfacial reactions in aqueous zinc batteries. Recent studies have demonstrated that modifying the coordination environment of Zn^2+^ through cosolvents or multifunctional electrolyte components can substantially reconstruct both the primary solvation sheath and the associated hydrogen‐bond network [46]. The reconstruction is quantitatively supported by the radial distribution functions (RDFs) and corresponding coordination numbers (CNs) (Figure 3b,c). At the first‐shell boundary of ∼0.25 nm, the H_2_O coordination number decreases from approximately 5.5 in the Zn(OAc)_2_ electrolyte to ∼4.7 in ZEFW2, accompanied by a reduction in OAc^−^ coordination from ∼0.9 to ∼0.6. Meanwhile, FA contributes a coordination number of ∼0.6 within the same region, whereas EtOH contributes only ∼0.2 and therefore has a relatively limited contribution to the innermost solvation shell. Importantly, the total coordination environment is not simply expanded by introducing additional solvent species; instead, the ligand population is redistributed among H_2_O, OAc^−^, FA, and EtOH. In particular, the emergence of FA coordination together with the reduced contributions from H_2_O and OAc^−^ provides direct molecular‐level evidence for reconstruction of the Zn^2+^ solvation structure. The time‐resolved RDF/CN evolution during the initial picosecond regime further demonstrates that these local coordination environments are established rapidly (Figures S8 and S9). Similar use of RDFs and coordination‐number analysis has been widely employed to identify changes in Zn^2+^ solvation structures induced by electrolyte engineering [47, 48]. The reconstructed solvation structure is accompanied by substantial reorganization of the intermolecular hydrogen‐bond network (Figure 3d,e). Compared with the highly interconnected H_2_O‐H_2_O network in the Zn(OAc)_2_ electrolyte, ZEFW2 exhibits a markedly reduced H_2_O‐H_2_O hydrogen‐bond population together with the emergence of extensive FA‐H_2_O and EtOH‐H_2_O interactions. These results indicate that the hydrogen‐bond network is redistributed rather than simply eliminated: the water‐dominated network characteristic of the conventional aqueous environment is partially replaced by mixed solvent‐water interactions. This behavior is consistent with recent reports showing that electrolyte components capable of strongly interacting with water can reorganize the hydrogen‐bond network and alter water activity and proton‐transfer pathways in aqueous Zn systems [47]. Consequently, the hydrogen‐bond restructuring provides a molecular basis for the altered ligand competition around Zn^2+^ and further supports the reconstructed solvation environment identified by the RDF/CN analysis. The effect of this molecular restructuring on Zn^2+^ transport was subsequently evaluated from the mean‐square displacement (MSD) obtained from the MD trajectories (Figure 3f). The slopes of the linear diffusive regions are 48.46 × 10^−3^ and 55.25 × 10^−3^ Å^2^ ps^−1^ for the Zn(OAc)_2_ and ZEFW2 electrolytes, respectively. Thus, despite the incorporation of additional coordinating species into the electrolyte, the reconstructed solvation environment does not result in a reduction in the simulated Zn^2+^ diffusivity; instead, a moderate increase of approximately 14% is obtained for ZEFW2. This result indicates that the redistribution of the primary solvation environment is compatible with Zn^2+^ transport and provides a molecular‐level basis for the favorable ion‐transport characteristics of ZEFW2. The combination of solvation restructuring and maintained Zn^2+^ transport is particularly relevant because electrolyte solvation structure can simultaneously influence ion mobility and the subsequent desolvation/interfacial reaction process [49].

**FIGURE 3:**
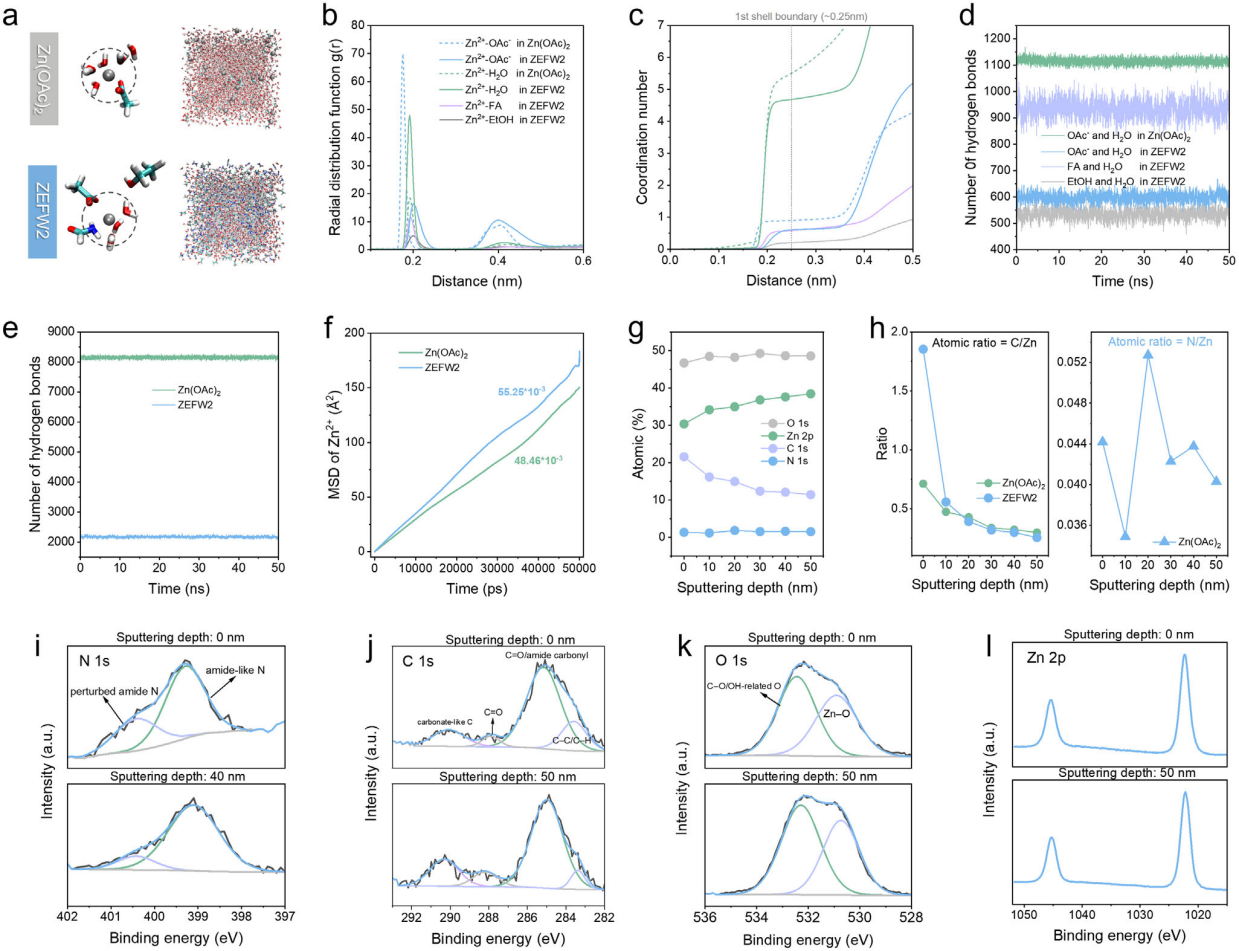
(a) Representative molecular configurations of the Zn^2+^ solvation environments in Zn(OAc)_2_ (upper panel) and ZEFW2 (lower panel) electrolytes. (b, c) Radial distribution functions and coordination numbers of Zn^2+^ in Zn(OAc)_2_ and ZEFW2 electrolytes. Time‐dependent evolution of intermolecular hydrogen bonds in Zn(OAc)_2_ and ZEFW2 electrolytes. (d) Hydrogen bonds between electrolyte components. (e) H_2_O‐H_2_O hydrogen bonds. (f) Mean‐square displacement and diffusion behavior of Zn^2+^ in Zn(OAc)_2_ and ZEFW2 electrolytes. (g) Depth‐dependent atomic percentages of N, C, O, and Zn obtained from XPS analysis of the ZEFW2‐derived Zn anode interphase. (h) Depth‐dependent C/Zn and N/Zn ratios derived from the XPS elemental compositions. (i–l) High‐resolution N 1s, C 1s, O 1s, and Zn 2p XPS spectra of the ZEFW2‐derived Zn interphase at nominal sputtering depths of 0 and 50 nm.

To determine whether the reconstructed bulk solvation environment is accompanied by a distinct chemical reconstruction at the Zn/electrolyte interface, depth‐resolved XPS measurements were performed on Zn anodes after 50 cycles in ZEFW2 (Figure 3g–l). Sequential Ar^+^ sputtering was used to probe the interfacial composition at nominal depths of 0, 10, 20, 30, 40, and 50 nm. The corresponding atomic percentages of Zn, O, C, and N are summarized in Figure 3g and Tables S2 and S3. At the outermost surface, the ZEFW2‐derived Zn interphase contains 30.37 at.% Zn, 46.69 at.% O, 21.60 at.% C, and 1.34 at.% N. With increasing sputtering depth, the Zn fraction gradually increases to 38.44 at.%, whereas the C fraction decreases to 11.44 at.% at 50 nm. Importantly, N remains detectable throughout the analyzed region, with an atomic concentration of approximately 1.2–1.8 at.%. These depth profiles demonstrate that the ZEFW2‐derived interphase contains persistent N‐ and C‐containing electrolyte‐derived species together with substantial Zn‐ and O‐containing components. The relative elemental ratios provide further quantitative insight into the interfacial composition (Figure 3h). Most notably, the C/Zn ratio decreases from ∼0.71 at the outermost surface to ∼0.30 at a nominal depth of 50 nm, indicating that the carbon‐rich component is most pronounced near the outer interfacial region and progressively approaches the underlying Zn composition with increasing depth. In contrast, the N/Zn ratio remains within approximately 0.035–0.053 throughout the analyzed region, with moderate point‐to‐point fluctuations but no monotonic depth dependence. We therefore interpret the N/Zn profile primarily as evidence for the persistence of N‐containing species across the analyzed interfacial region rather than as an indicator of progressive N enrichment or depletion [50, 51]. The chemically more informative depth evolution is instead revealed by the N 1s peak‐area redistribution discussed below.

High‐resolution XPS provides further information on the chemical environments of the interfacial species (Figure 3i–l). The N 1s spectra contain two distinguishable components at approximately 399.3 and 400.5 eV at the analyzed depths. The lower‐binding‐energy component is associated with an amide‐like/Zn‐coordinated N‐containing environment, whereas the higher‐binding‐energy component corresponds to a higher‐binding‐energy amide‐related N environment. Their relative contributions evolve systematically with depth: the 399.3 eV component increases from 67.75% at the outermost surface to 86.62% at a nominal depth of 40 nm, whereas the 400.5 eV component decreases from 32.25% to 13.38%. Thus, although the total N concentration does not exhibit a monotonic depth dependence, its chemical‐state distribution changes systematically across the analyzed interfacial region. This distinction between elemental abundance and chemical‐state evolution is important for identifying the chemically heterogeneous nature of the ZEFW2‐derived interphase [52]. The C 1s spectra further reveal a depth‐dependent redistribution of carbon‐containing chemical environments. Four components centered at approximately 284, 285.5, 288, and 290.3 eV are resolved and assigned to C─C/C─H, C─C/C─N‐rich, carbonyl/amide‐related, and higher‐binding‐energy oxidized carbon environments, respectively. From the surface to 50 nm, the contribution of the 284 eV component decreases from 17.42% to 6.39%, whereas the carbonyl/amide‐related component increases from 4.35% to 8.70%. The dominant 285.5 eV component remains comparatively stable, while the high‐binding‐energy component also increases with depth [53]. These changes indicate that the carbon‐containing species are chemically redistributed within the interphase rather than simply forming a uniform organic coating. The O 1s spectra contain a lower‐binding‐energy component at approximately 530.9 eV, attributed primarily to Zn─O‐related oxygen, and a higher‐binding‐energy component near 532.4 eV associated with C─O/OH‐related oxygen environments. Their relative contributions remain comparatively stable with depth, with the Zn‐O‐related component changing from 45.60% at the surface to 43.54% at 50 nm. The persistence of this component supports the presence of Zn─O‐containing inorganic species throughout the analyzed interfacial region. Meanwhile, the Zn 2p spectra show essentially unchanged Zn 2p_3/2_ and Zn 2p_1/2_ features with increasing sputtering depth, indicating that Zn remains predominantly in the Zn^2+^ state [54]. Because individual XPS components can involve overlapping chemical environments, the XPS data are used to establish elemental composition and chemical‐state evolution rather than to assign absolute phase fractions of specific compounds.

The MD and XPS results establish a continuous molecular‐to‐interfacial picture for ZEFW2. The incorporation of FA and EtOH redistributes the ligands within the Zn^2+^ solvation shell, reorganizes the hydrogen‐bond network, and maintains favorable Zn^2+^ transport. This reconstructed electrolyte environment is accompanied by the formation of a chemically distinct Zn interphase containing persistent N‐ and C‐containing electrolyte‐derived species together with Zn‐O‐related inorganic components.

### 2.3 Zn Nucleation Regulation

By altering the Zn^2+^ solvation sheath and interfacial chemistry, ZEFW2 fundamentally modulates Zn nucleation thermodynamics and deposition morphology. In‐situ optical microscopy was employed to directly monitor Zn plating dynamics in real time (Figure 4a). In Zn(OAc)_2_ electrolyte, surface protrusions appeared within just 10 min at 4 mA cm^−2^, quickly evolving into bulky dendrites as plating continued. By contrast, ZEFW2 maintained a smooth, uniform interface throughout 60 min of continuous deposition, confirming its ability to promote homogeneous Zn^2+^ flux and suppress dendrite initiation from the earliest stages of growth. AFM analysis was conducted to visualize the post‐cycling surface topography (Figure 4b). In the baseline Zn(OAc)_2_ electrolyte at 25°C, the Zn surface was covered with pronounced dendritic protrusions and deep trench‐like grooves, yielding a high root‐mean‐square roughness (R_q_) of 70.5 nm (Figures S10 and S11). Introducing ZEFW2 dramatically flattened the surface, reducing R_q_ to 46.5 nm at 25°C and further to 25.4 nm at −20°C, hinting at superior interfacial stability under low‐temperature conditions (Figure S12). SEM imaging provided direct evidence of the deposition quality (Figure 4c and Figure S13). After cycling in Zn(OAc)_2_, Zn electrodes exhibited mossy, loosely packed deposits, whereas ZEFW2 produced compact, hexagonally stacked deposits. Even at −20°C, ZEFW2 maintained dense and uniform Zn layers, underscoring its robustness for low‐temperature zinc metal batteries, which were consistent with AFM observations (Figure S14). Since electrolyte‐electrode interactions play a decisive role in nucleation uniformity, the wettability of ZEFW2 on both Zn and NaV_3_O_8_·1.5H_2_O (NVO) electrodes was characterized (Figure 4d). ZEFW2 exhibited markedly smaller contact angles (51.5° on Zn and 47.7° on NVO) compared to Zn(OAc)_2_ (88.0° and 74.4°), indicating enhanced surface wetting that favors uniform Zn^2+^ distribution and more consistent nucleation sites across the electrode surface [55]. To understand the origin of the preferential (101) facet growth, density functional theory (DFT) calculations were performed to evaluate the adsorption behavior of EF and H_2_O molecules on the dominant Zn surfaces (Figure 4e). The fully optimized adsorption configurations of H_2_O, ethanol, FA, and EF on the Zn(002), Zn(100), and Zn(101) surfaces, together with the corresponding Zn^2+^ adsorption configurations on pristine and EF‐modified surfaces, are provided in Figures S15–S20, while the calculated adsorption energies are summarized in Table S4. The calculated adsorption energies (E_ads_) of EF on the (002), (100), and (101) facets are consistently more negative than those of H_2_O, indicating that EF molecules interact more strongly with Zn surfaces and can effectively compete with water at the interface. Notably, EF exhibits the most favorable adsorption on the (101) surface (−1.17 eV), compared with −0.90 eV on (100) and −0.88 eV on (002), suggesting that EF adsorption preferentially stabilizes this facet. Structural analysis shows that the interaction is primarily mediated by polar O‐containing groups anchoring to surface Zn atoms, which modulates the local coordination environment and surface energetics. Furthermore, the adsorption of Zn^2+^ on pristine and EF‐modified Zn surfaces was examined (Figure 4f). The EF‐modified interface provides a more favorable energetic landscape for Zn^2+^ accommodation, lowering the nucleation barrier on the (101) facet and thereby promoting its dominant growth.

**FIGURE 4:**
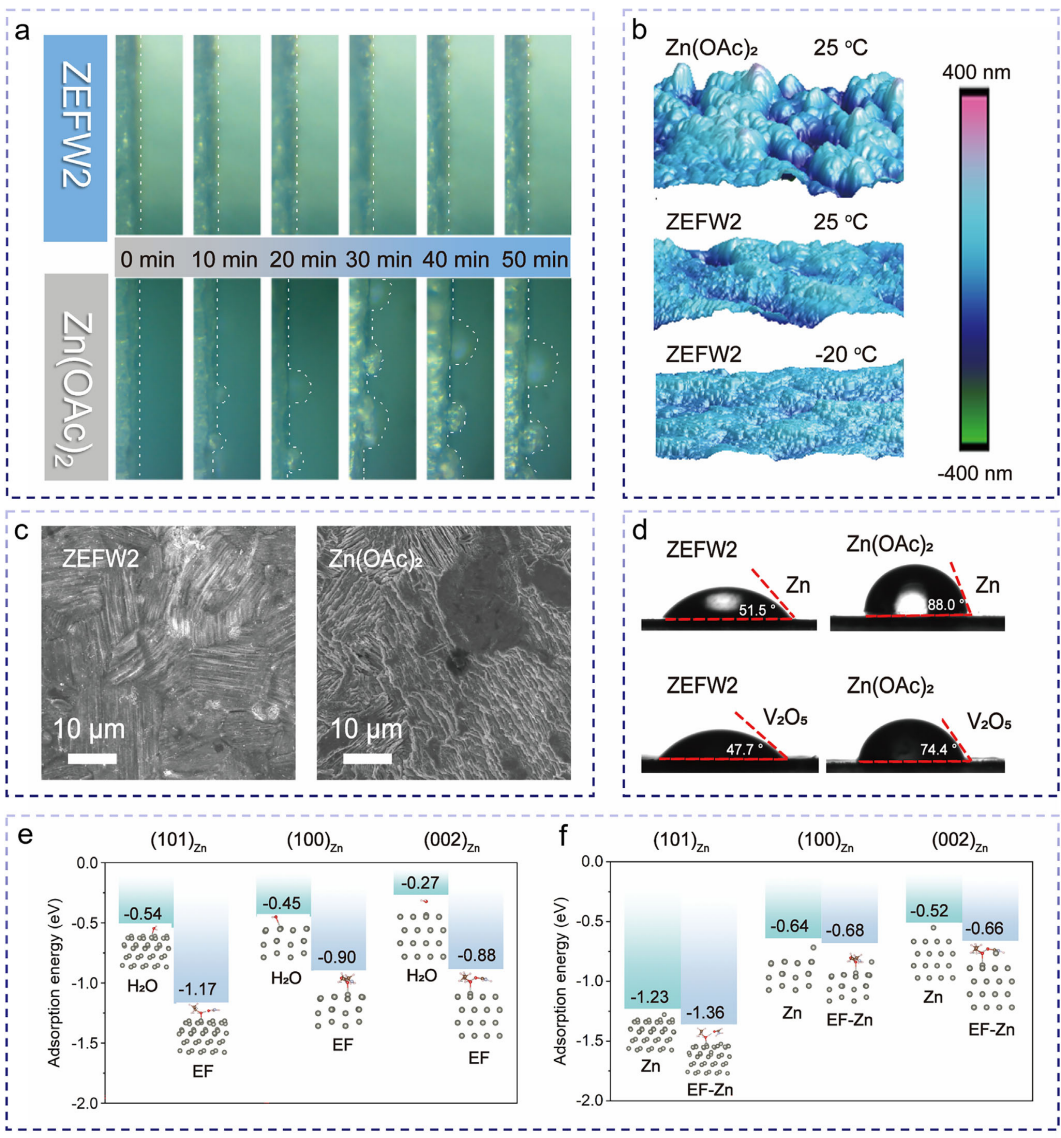
(a) In situ optical microscopic observation of dendrite growth with Zn(OAc)_2_ and ZEFW2 at 4 mA cm^−2^ at 25°C. (b) AFM images of Zn anodes after 100 cycles for different electrolytes at different temperatures. (c) SEM images of the Zn metal surface in Zn||Zn cells after 20 cycles with Zn(OAc)_2_ and ZEFW2 at 25°C. (d) Contact angle measurement on Zn foil and NVO electrodes at 25°C. (e) DFT‐calculated adsorption energy of H_2_O or EF on Zn. (f) DFT‐calculated adsorption energy of Zn^2+^ on Zn foil or Zn foil adsorbed with EF.

### 2.4 Facet‐Preferred Zn Deposition

The crystallographic origins of the smoother Zn deposition in ZEFW2 were further probed via XRD tracking of Zn deposits at 25°C and −20°C (Figures S21 and S22). In Zn(OAc)_2_, the (101) facet fraction gradually decreased during cycling, whereas ZEFW2 promoted its continuous growth. At −20°C, the (101) facet developed even more rapidly, with its fraction increasing from 35% to 68% after 100 cycles (vs. decreasing to 22% in Zn(OAc)_2_), consistent with the exceptionally smooth surfaces seen in AFM and SEM. These results indicate that the EF‐based deep eutectic electrolyte induces preferential (101) facet formation, which guides uniform Zn^2+^ deposition. To probe the atomic‐scale origins of this facet‐selective behavior, Zn K‐edge XANES and EXAFS characterizations were performed (Figure 5). In the pristine state, the formamide‐based ZEFW2 yielded a Zn valence state of approximately +1.7, markedly lower than the fully oxidized +2 state in the Zn(OAc)_2_ system (close to the ZnO standard, Figure 5a). EXAFS fitting confirms that ZEFW2 exhibits a significant Zn‐N coordination number, whereas the Zn(OAc)_2_ system shows negligible Zn‐N bonding (Tables S5 and S7). This partial reduction originates from the strong electron‐donating ability of the formamide nitrogen atom, whose lone pair electrons are injected into the Zn 4s orbital to form a partially reduced [Zn(^δ^^+^)‐N] coordination structure (δ ≈ +1.7) [56, 57]. By contrast, Zn(OAc)_2_ forms fully oxidized Zn^2+^‐OCO coordination. After cycling, ZEFW2 exhibited a further valence decrease to +1.5 (Figure 5b), with EXAFS analysis indicating an increase in both Zn‐N and unavoidable Zn‐O coordination numbers, reflecting the formation of mixed Zn clusters and the retention of Zn‐N species during cycling (Tables S6 and S8). In contrast, Zn(OAc)_2_ maintained a high oxidation state throughout cycling, consistent with the continuous accumulation of a carbonate passivation layer. The edge shift to higher energy in ZEFW2 can be attributed to electron loss and formation of new Zn‐N chemical bonds (Figure 5c,d). EXAFS analysis in R‐space demonstrated a distinct contrast between ZEFW2 and Zn(OAc)_2_ (Figure 5e) [58]. For ZEFW2, the Zn‐N/O peak at 1.58 Å was significantly more intense than the Zn‐Zn peak at 2.37 Å, clearly highlighting the dominant role of Zn‐N coordination in suppressing the formation/exposure of bulk‐like Zn species. In contrast, the spectrum of Zn(OAc)_2_ exhibited shorter Zn‐O (1.48 Å) and Zn‐Zn (2.18 Å) distances, along with a discernible shoulder at 1.82 Å, consistent with Zn‐O‐C bonding within carbonate‐rich passivation layers [59]. Cycling in ZEFW2 caused the Zn‐Zn peak to split into two components (2.07 and 2.44 Å) (Figure 5f,g and Figure S23), reflecting the coexistence of nanoscale Zn clusters and bulk Zn, and the shared 3.03 Å peak was well‐fitted by a Zn‐phthalocyanine (Zn‐Pc) model, confirming the stable retention of organic ligands during cycling. In Zn(OAc)_2_, peak positions remained similar, but the Zn‐Zn intensity increased, consistent with interface disorder from carbonate build‐up [60, 61]. Wavelet transform (WT) analysis provides complementary k‐R space resolution to further elucidate the dynamic Zn coordination environments (Figure 5h–k). Reference WT contour plots of Zn foil, ZnO, Zn_5_(OH)_6_(CO_3_)_2_, and Zn‐Pc (Figure S24) establish characteristic fingerprints for Zn‐Zn metallic bonding and Zn‐O/N coordination, enabling unambiguous phase discrimination. For ZEFW2, two well‐separated intensity maxima at (5.78 Å^−1^, 1.65 Å) and (7.33 Å^−1^, 2.57 Å) are assigned to low‐k Zn‐N coordination associated with the dynamic solvation shell and high‐k Zn‐Zn metallic bonding from the bulk lattice, respectively. After cycling, the Zn‐N k‐value increases to 5.96 Å^−1^ and the Zn‐Zn R‐value expands to 2.64 Å, indicating strengthened coordination interactions and lattice relaxation during uniform Zn deposition. In contrast, Zn(OAc)_2_ exhibits initial WT maxima at (4.97 Å^−1^, 1.57 Å) and (7.34 Å^−1^, 2.27 Å), which, after cycling, shift toward lower k (4.85 Å^−1^) and smaller R (2.19 Å), reflecting bond weakening and lattice compression‐spectroscopic hallmarks of dendritic growth. Notably, the ∼0.9 Å^−1^ separation in the low‐k region between ZEFW2 (∼5.8 Å^−1^) and Zn(OAc)_2_ (∼4.9 Å^−1^) serves as a robust WT fingerprint for distinguishing Zn─N from Zn‐OAc coordination, further corroborating the solvation‐regulated deposition mechanism.

**FIGURE 5:**
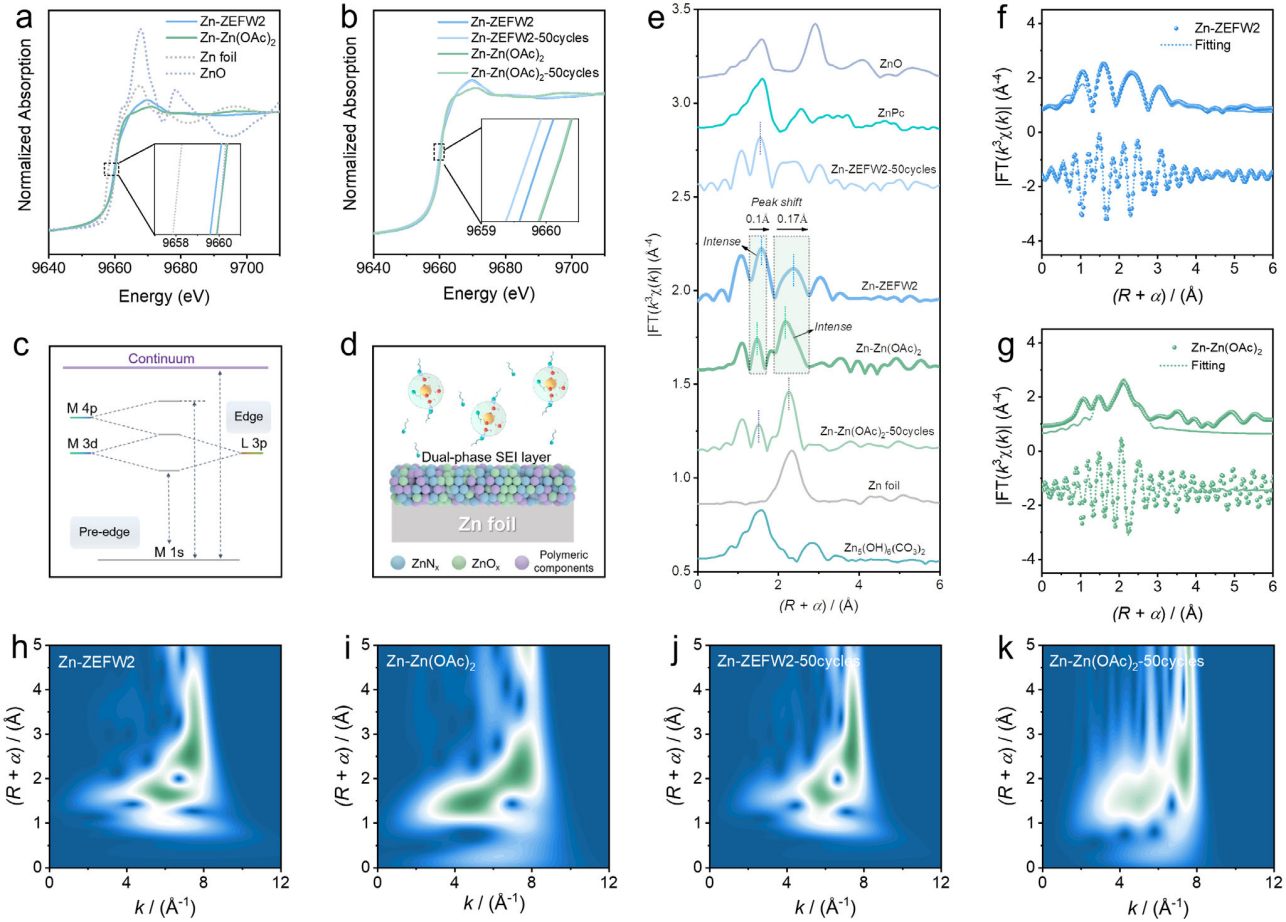
(a) The normalized Zn K‐edge XANES spectra of Zn foil soaked in Zn(OAc)_2_ and ZEFW2. (b) The normalized Zn K‐edge XANES spectra before and after cycling in the Zn(OAc)_2_ and ZEFW2 at 25°C. (c) Diagram of electron transition. (d) Diagram of ZEFW2 adsorption on Zn anode. (e) FT‐EXAFS spectra in R space. (f,g) The fitted FT‐EXAFS spectra in R space. (h,k) Wavelet transform images of the EXAFS spectra.

### 2.5 Hybrid Interphase Formation

The reconstructed solvation structure drives in‐situ formation of a hybrid SEI, acting as a bridge between solvation regulation and crystallographic orientation control. The strong Zn─N coordination in ZEFW2 not only modulates the solvation environment but also promotes the adsorption of eutectic molecules on the Zn surface, inducing in‐situ formation of a robust hybrid SEI. EDS mapping detected N species on the ZEFW2‐cycled Zn surface, providing direct evidence of EF adsorption and participation in SEI formation. Combined with XAS Zn K‐edge analysis (Figure 5a,b), the SEI is identified as a dual‐phase structure composed of inorganic (ZnO, ZnN) and organic (polymerized FA/EtOH derivatives) components. The partial electron redistribution around Zn centers in ZEFW2 indicates formation of Zn─N bonds in the interfacial layer, further supporting the presence of Zn‐N species in the SEI. This hybrid SEI significantly enhances interfacial stability, as demonstrated by electrochemical impedance spectroscopy (EIS) of Zn‐NVO full cells (Figure S25) and symmetric Zn||Zn cells (Figure S26). ZEFW2 exhibited the lowest charge‐transfer resistance (R_ct_ = 85 Ω at 25°C, 156 Ω at −20°C) among tested electrolytes, indicating accelerated interfacial charge transfer. Tafel plots (Figure S27) revealed that ZEFW2 possessed the most positive corrosion potential (−0.78 V vs. SHE) and the lowest corrosion current (0.21 mA cm^−2^ at 25°C, 0.15 mA cm^−2^ at ‐20°C), highlighting its enhanced anti‐corrosion effect and improved Zn^2+^ kinetics [62]. Additionally, linear sweep voltammetry (LSV) (Figure S28) demonstrated that ZEFW2 maintained a significantly wider electrochemical stability window (1.8 V at 25°C, 1.9 V at −20°C) than Zn(OAc)_2_ (1.6 V at 25°C, 1.5 V at −20°C), which further broadened under subzero conditions, implying more effective suppression of hydrogen and oxygen evolution [63]. The stability of the SEI is further supported by the low fraction of strongly hydrogen‐bonded water in ZEFW2 (19.2%), which reduces water‐induced SEI decomposition. This explains why ZEFW2 maintains constant charge‐transfer resistance during cycling, while Zn(OAc)_2_ shows increased resistance due to SEI degradation. The enhanced wettability of ZEFW2 (Figure 4d) also contributes to SEI uniformity, because uniform electrolyte spreading facilitates consistent SEI formation across the electrode surface. Cyclic voltammetry (CV) measurements indicated the largest nucleation overpotential in ZEFW2 (Figure S29), favoring smaller nuclei and more homogeneous deposition [64]. Chronoamperometry confirmed a rapid transition from short 2D to uniform 3D growth in ZEFW2, in contrast to the persistent disordered 2D growth in Zn(OAc)_2_ (Figures S30 and S31). Overall, the preferential (101) facet formation in ZEFW2 enables uniform Zn^2+^ nucleation, broadens the stability window, suppresses side reactions, and delivers superior low‐temperature electrochemical performance.

### 2.6 Electrochemical Validation

The triple‐coupling regulation endows ZEFW2 with exceptional electrochemical performance at both room and subzero temperatures. Zn||Zn symmetric cells were assembled for galvanostatic cycling and rate capability tests at 25°C. As shown in Figure 6a, the cell with ZEFW2 electrolyte exhibited stable cycling for over 1000 h at 0.5 mA cm^−2^ with an areal capacity of 0.25 mAh cm^−2^, while the Zn(OAc)_2_ electrolyte cell failed within 50 h under identical conditions. Other deep eutectic formulations fail at 400–600 h under the same conditions, illustrating the importance of specific deep eutectic formulations (Figure S32). Rate capability tests from 0.25 to 4 mA cm^−2^ (Figure S33) revealed that Zn(OAc)_2_ cells suffered severe polarization at high currents, whereas ZEFW2 cells maintained stable voltage profiles. At −20°C, ZEFW2‐based symmetric cells operated stably for over 1400 h at 1 mA cm^−2^ with 10 mAh cm^−2^ deposition (Figure 6b) and nearly 2000 h at 0.5 mA cm^−2^/0.5 mAh cm^−2^ (Figure S34), with robust rate performance (Figure S35), confirming that fast Zn^2+^ plating/stripping kinetics are preserved even under subzero conditions, consistent with the reconstructed solvation structure and robust interfacial chemistry. The Coulombic efficiency (CE) of Zn plating/stripping in Zn||Cu cells further highlights the electrolyte's advantage. At 25°C and 1 mA cm^−2^/0.25 mAh cm^−2^, ZEFW2 cells achieved a CE of 99.44% after 961 cycles, compared to only 84.99% after 31 cycles for Zn(OAc)_2_ cells (Figure 6c). Even at −20°C, ZEFW2 maintained 99.78% CE over 1200 cycles (Figure 6d), indicating greatly improved reversibility. CV analysis (Figure 6e,f) confirmed excellent reversibility at both temperatures with ZEFW2, but Zn(OAc)_2_ cells show poor cyclic stability (Figure S36) [65]. The higher cumulative CV area (Figure S37) indicated faster reaction kinetics with ZEFW2 [66]. Full‐cell tests with NVO cathodes demonstrated the practical applicability of ZEFW2. At 25°C, capacities of 310, 250, and 226 mAh g^−1^ were delivered at 1, 2, and 3 A g^−1^, which were always higher than those of the Zn(OAc)_2_ (270 mAh g^−1^ at 1 A g^−1^, 219 mAh g^−1^ at 2 A g^−1^, and 196 mAh g^−1^ at 3 A g^−1^). Rate performance comparisons at room temperature (Figure S38) directly demonstrate that ZEFW2 facilitates faster Zn^2+^ transfer and more efficient utilization of the NVO cathode, which originates from the weakened synergistic solvation sheath that promotes Zn^2+^ participation in the redox process. At −20 °C, capacities of 181, 156, and 112 mAh g^−1^ were obtained at 1, 2, and 3 A g^−1^, showing an admirable discharge capacity and no loss upon current recovery. The robust low‐temperature rate capability is further confirmed in Figure S39, evidencing sustained Zn^2+^ transport and interfacial stability under harsh subzero conditions. Long‐term cycling at 25°C (3 A g^−1^) yielded 61% capacity retention after 1800 cycles for ZEFW2, versus 31% after 700 cycles for Zn(OAc)_2_ (Figure 6g). As a demonstration of practical application, two Zn||NVO pouch cells applying ZEFW2 electrolyte were connected in series to power a light‐emitting diode (LED) light (Figure S40). At 1 A g^−1^, ZEFW2 retained 65% capacity after 150 cycles, versus 32% after 81 cycles for Zn(OAc)_2_ (Figure S41). At −20 °C and 1 A g^−1^, ZEFW2 retained 74% capacity after 1600 cycles (Figure 6h). Notably, at −20°C, the cumulative capacity of the ZEFW2 surpasses many previously reported electrolytes (Figure 6i). A Zn‐NVO pouch cell at 25°C and 0.5 A g^−1^ retained 61% capacity after 500 cycles (Figure S42), emphasizing its practical application value. A multidimensional comparison of cycling time, areal capacity, and current density was conducted at −20°C (Figure 6j), these results place ZEFW2 in a distinct high‐capacity and long‐lifetime region of the low‐temperature performance map, demonstrating robust Zn reversibility across different operating conditions, the corresponding data are summarized in Table S9.

**FIGURE 6:**
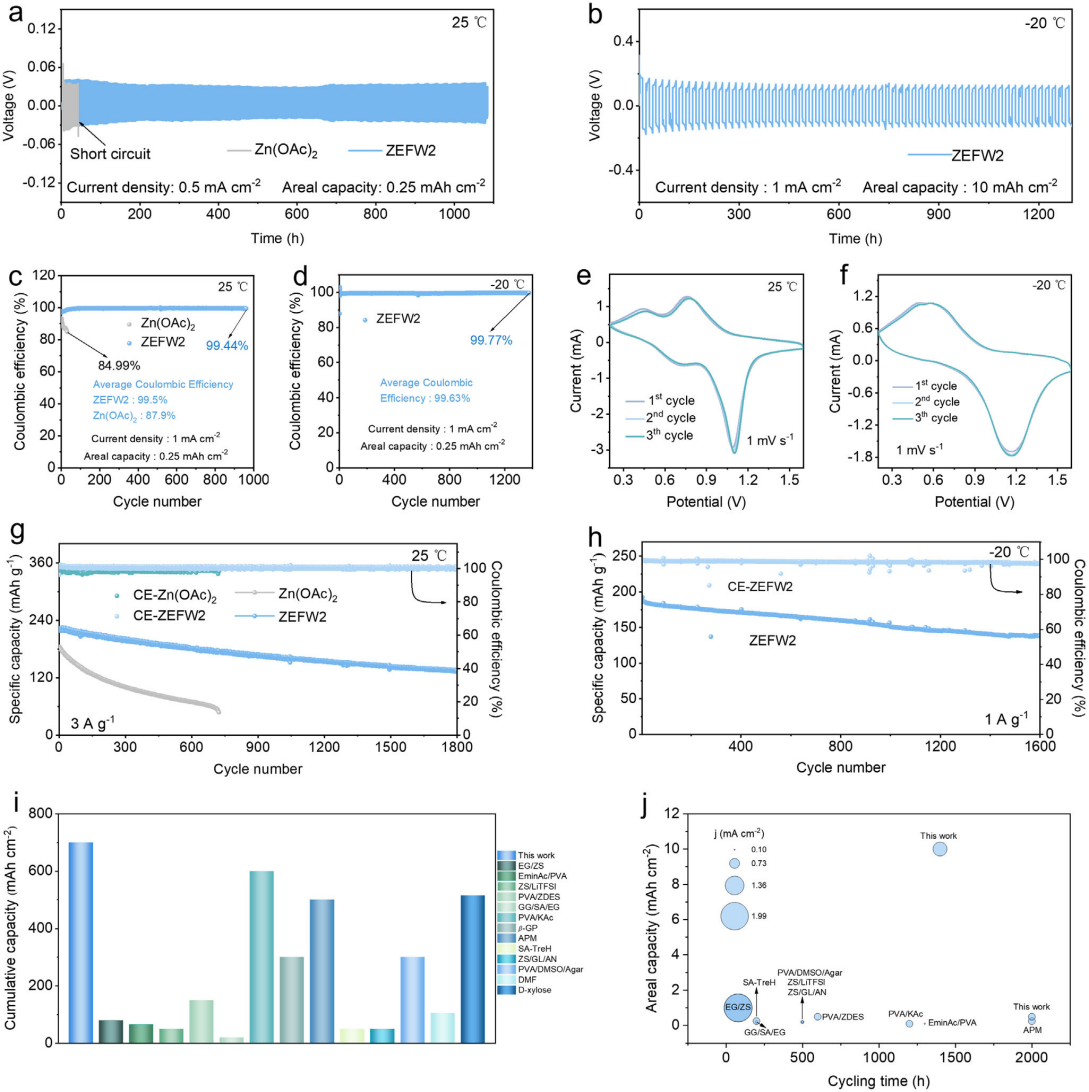
(a) Cycling performance of the Zn||Zn cells with different electrolytes at a current density of 0.5 mA cm^−2^ and an areal capacity of 0.25 mAh cm^−2^ at 25°C. (b) Cycling performance of the Zn||Zn cell with ZEFW2 at a current density of 1 mA cm^−2^ and an areal capacity of 10 mAh cm^−2^ at −20°C. The CE of Zn plating/stripping in Zn||Cu cells with ZEFW2 at a current density of 1 mA cm^−2^ and an areal capacity of 0.25 mAh cm^−2^. (c) at 25°C. (d) at −20°C. CV curves of Zn||NVO cell operated at different cycles with ZEFW2 at 1 mV s^−1^ (e) at 25°C and (f) at −20°C. (g) Long‐term cycling performance of Zn||NVO cells at 3 A g^−1^ at 25°C. (h) Long‐term cycling performance of Zn||NVO cell at 1 A g^−1^ at −20°C. (i) Cumulative capacity comparison between this work and recent studies at −20°C. (j) Multidimensional comparison of ZEFW2 and previously reported electrolytes at −20°C. The cycling time, areal capacity, and current density are represented by the x‐axis, y‐axis, and bubble size, respectively.

### 2.7 Practical Pouch‐Cell Validation

To further evaluate the practical applicability of the ZEFW2 electrolyte, pouch cells with a size of 5 × 4.5 cm^2^ were assembled and evaluated under both subzero and room‐temperature conditions (Figure S43). The pouch‐cell configuration and physical dimensions are shown in Figure S15a,b and Table S10. At −20°C, the ZEFW2‐based pouch cell remained electrochemically operable and exhibited well‐defined voltage‐specific capacity profiles over prolonged cycling (Figure S44a). After an initial conditioning period, the discharge capacity stabilized at approximately 0.0043–0.0046 Ah and gradually decreased to 0.00418 Ah after 406 cycles, corresponding to ∼92% retention relative to the stabilized early‐cycle capacity (Figure S44b). The largely preserved voltage profiles further indicate that the cell maintained its overall electrochemical characteristics during subzero cycling. The room‐temperature pouch‐cell performance was further examined by comparing ZEFW2 with the Zn(OAc)_2_ electrolyte. As shown in Figure S45a,b, the voltage‐specific capacity profiles of the ZEFW2‐based pouch cell remained measurable over extended cycling, whereas the Zn(OAc)_2_‐based cell exhibited progressively diminished capacity with cycling. Consistently, the discharge‐capacity evolution in Figure S45c shows a substantially slower capacity decay for ZEFW2 than for Zn(OAc)_2_. The ZEFW2‐based cell retained a measurable capacity over 3000 cycles, while the capacity of the Zn(OAc)_2_‐based cell decreased to a much lower level. In addition, repeated attempts to operate the Zn(OAc)_2_‐based pouch cell at −20°C were unsuccessful because the electrolyte solidified under the test conditions, preventing stable electrochemical operation. Together, these pouch‐cell results provide practical‐level evidence that the ZEFW2 electrolyte maintains electrochemical functionality under both room‐temperature and subzero conditions.

The superior low‐temperature performance of ZEFW2 originates from a coherently integrated triple‐coupling regulation of Zn^2+^ solvation, interfacial chemistry, and crystallographic deposition behavior. The reconstructed solvation environment simultaneously accelerates Zn^2+^ transport and suppresses electrolyte freezing, while the resulting hybrid SEI stabilizes the Zn/electrolyte interface by mitigating corrosion and parasitic side reactions. In parallel, the induced (101) facet‐preferred Zn deposition ensures homogeneous metal growth and effective dendrite suppression. Acting synergistically, these coupled effects overcome the intrinsic kinetic and interfacial constraints of aqueous Zn metal anodes under subzero conditions, underscoring triple‐coupling regulation as a generalizable strategy for designing temperature‐resilient metal‐ion batteries.

## 3 Conclusion

In summary, a triple‐coupling regulation strategy integrating solvation structure, interfacial chemistry, and crystallographic orientation to improve the low‐temperature performance of aqueous Zn‐ion batteries was established. Molecular dynamics simulations demonstrate that incorporating formamide and ethanol redistributes the primary Zn^2+^ solvation environment, reducing H_2_O and acetate coordination while introducing formamide coordination and reorganizing the hydrogen‐bond network without compromising Zn^2+^ transport. Depth‐resolved XPS reveals that this reconstructed solvation environment is accompanied by a chemically reconstructed Zn interphase containing Zn‐O‐related inorganic species and persistent N‐ and C‐containing electrolyte‐derived species. Together with structural and theoretical analyses, these results establish a continuous molecular‐to‐interfacial regulation pathway. The modified interfacial environment regulates Zn nucleation and promotes preferential Zn deposition along the (101) facet, leading to more homogeneous Zn growth and suppressed dendritic morphology. As a result, Zn||Zn symmetric cells achieve 1400 h of cycling at −20°C under 1 mA cm^−2^ and 10 mAh cm^−2^, with 99.78% Coulombic efficiency over 1200 cycles. Zn||NVO full cells retain 74% of their initial capacity after 1600 cycles at −20°C and maintain stable operation over a broad temperature range. Multidimensional benchmarking further demonstrates the combination of high areal capacity, relatively high current density, and prolonged cycling durability. Pouch‐cell tests provide additional validation of stable electrochemical operation at both room temperature and −20°C. Overall, this work provides a molecular‐to‐interfacial design framework for developing temperature‐resilient AZIBs and offers a general strategy for regulating Zn deposition under challenging low‐temperature conditions.

## Author Contributions

**Mengyang Dong**: conceptualization, methodology, software, data curation, investigation, writing – original draft, writing – review and editing, visualization. **Zhibao Yang**: conceptualization, software, data curation, formal analysis, validation, visualization, writing – original draft. **Lina Pan**: conceptualization, writing – review and editing, validation, visualization, software, formal analysis, data curation. **Jie Qu**: conceptualization, methodology, data curation, resources, supervision, formal analysis, funding acquisition, writing – review and editing, investigation. **Shangshu Qian**: investigation, validation, software, visualization, data curation. **Jessica Hamilton**: conceptualization, software, data curation, funding acquisition. **Bernt Johannessen**: conceptualization, software, data curation, funding acquisition. **Yuhui Tian**: conceptualization, investigation, writing – review and editing, visualization, validation, software, data curation. **Chao Xing**: conceptualization, methodology, software, data curation, supervision, resources, writing – review and editing, writing – original draft, investigation, funding acquisition.

## Conflicts of Interest

The authors declare no conflicts of interest.

## Supporting information




**Supporting File**: advs78147‐sup‐0001‐SuppMat.docx.

## Data Availability

The data that support the findings of this study are available from the corresponding author upon reasonable request.
