# Supplementary material for: Triple‐Coupling Regulation Enables Dendrite‐Free Subzero Aqueous Zn‐Ion Batteries
Source: Adv Sci (Weinh). 2026 Oct 8:e78147. Online ahead of print. doi: 10.1002/advs.78147 (PMC13650021; doi:10.1002/advs.78147)
Supplement: Supplementary file 1 [file ADVS-9999-e78147-s001.docx]

**Triple-Coupling Regulation Enables Dendrite-Free Subzero Aqueous Zn-Ion Batteries**

Mengyang Dong^1,2^, Zhibao Yang^1^, Lina Pan^1^, Jie Qu^1,^*, Shangshu Qian^3^, Jessica Hamilton^4^, Bernt Johannessen^4^, Yuhui Tian^5,^*, Chao Xing^2,^*

^1^ M. Dong, Z. Yang, L. Pan, J. Qu

College of Chemistry and Chemical Engineering, Hunan Normal University, Changsha, 410081, China

E-mail: [qujie@hunnu.edu.cn](mailto:qujie@hunnu.edu.cn)

^2^ M. Dong, C. Xing

UQ Dow Centre for Sustainable Engineering Innovation, School of Chemical Engineering, The University of Queensland, Queensland 4072, St Lucia, Australia

E-mail: c.xing@uq.edu.au

^3^ S. Qian

College of Chemical and Biological Engineering, Zhejiang University, Hangzhou 310058, China

^4^ J. Hamilton, B. Johannessen

Australian Synchrotron, ANSTO, Clayton, VIC 3168, Australia

^5^ Y. Tian

School of Molecular and Life Sciences, Curtin University, Bentley, Western Australia 6102, Australia

E-mail: yuhui.yhtian@gmail.com

Mengyang Dong, Zhibao Yang and Lina Pan contributed equally to this work and should be regarded as co-first authors.

Keywords: Aqueous zinc-ion batteries, triple-coupling regulation, solvation engineering, facet-preferred Zn deposition, hybrid interphase, subzero electrochemistry

## Experimental Part

*Chemicals*: V_2_O_5_ (≥99.5%, Aladdin), Zn(OAc)_2_ (AR, Macklin), NaCl (≥99.5%, Aladdin), Formamide (AR, Aladdin), Ethanol (AR, Greagent), Single-Wall Carbon Nano Tubes purchased from Shenyang Huijing Nanotech Co., Ltd, Zinc foil (CR2032) purchased from **Jinghong New Energy Co., Ltd., Cu foil (99.99%)** purchased from **Jingliang** **Copper Industry Co., Ltd., Glass Fiber Separator**, **Stainless Steel and** Ti foil (99.99%) purchased from **Xuzhou Chaoxin Energy Technology Co., Ltd.,**

*Preparation of NVO (NaV_3_O_8_·1.5H_2_O) and electrolytes:* Add 1g of V_2_O_5_ (≥99.5%, Aladdin) to a 2 M NaCl (≥99.5%, Aladdin) solution. Stir for 90 minutes at 25 ℃. The resulting solution is washed three times with deionised water and ethanol, then dried at 80 ℃ for 12 hours, recorded as NVO. Mix EtOH and FA according to the molar ratio of 1:1, 1:2, 1:4, heat and stir at 80 ℃ for 1 hour to prepare a mixed solution. The prepared mixed solution is combined with deionised water in a volume ratio of 6:4, and 1M Zn(OAc)_2_ (AR, Macklin) is added and stirred at 25°C for 1 hour, recorded as ZEFW1、ZEFW2、ZEFW4.

**Characterization.**

Powder X-ray diffraction (XRD) patterns were recorded on a D8 ADVANCE X-ray diffractometer using Cu Kα radiation (λ=1.54056 Å) within the 2θ range of 10-80°. Nuclear Magnetic Resonance (NMR) spectroscopy was performed on a DPX 400 MHz operated. Fourier-transform infrared spectroscopy (FTIR) was conducted on a VERTEX 80v instrument, covering a wavenumber range of 400-4000 cm^-1^. Scanning Electron Microscopy (SEM) was carried out using a Hitachi-SU8020 field emission. Differential scanning calorimetry was performed using a DSC 204 F1 Phoenix differential scanning calorimeter instrument within the temperature range of -80 to 25 °C. Thermogravimetric analysis (TGA) was performed using a 3+ Mettler Toledo thermogravimetric analyzer (Switzerland). The surface roughness of the negative electrode was measured using Dimension Icon Atomic Force Microscopy. The contact angle between the electrolyte and the electrode interface was measured using the JC2000D3M contact angle meter. This study used the LabRAM HR 800 Raman spectrometer produced by Horiba, France, to scan different electrolytes and analyse the hydrogen bond network characteristics in different electrolytes at -20 ^o^C and 25 ^o^C through peak fitting, with a scanning range of 400 cm^-1^ to 4000 cm^-1^. X-ray photoelectron spectroscopy (XPS) measurements were conducted using a Thermo Scientific K-Alpha X-ray photoelectron spectrometer, with a sputtering rate of 0.4 nm s^-1^ for depth-profile analysis. The X-ray absorption fine structure measurements were carried out using the hard X-ray spectroscopy beamline (XAS) of the Australian Synchrotron.

**Electrochemical measurements.**

0.8 g NVO and 0.2 g single-walled carbon nanotubes (SWCNT) were weighed in a mortar and ground for 40 min. After thorough mixing, the synthesized mixture was applied onto the Titanium foil and dried at 80℃ for 6 h. The loading mass of VON is approximately ~1 mg cm^−2^. The Zn||Zn symmetric cells, Cu||Zn asymmetric cells and NVO||Zn full cells were assembled into CR2032 coin cells. The cells were assembled by using 12 mm Zn plates as the anode and glass fiber as the separator. The electrochemical performance testing was conducted on a Neware (CT-4800) battery cycling system. Electrochemical characterizations, such as LSV, Tafel and CA et al., were performed on the electrochemical workstation (CHI604E, CHI660E. The LSV profiles were tested from -0.3 to -3 V with a scan rate of 10 mV s^−1^. The Tafel curves were measured from -0.3 to 0.3 V with a scan rate of 1 mV s^−1^. The chronoamperometry tests curves were performed at a constant potential of -0.15 V. Electrochemical impedance spectroscopy was tested within a frequency range of 100 kHz to 0.01 Hz. The scan rate of the CV test is 1 mV s^-1^. For in-situ optical microscopy measurement, two Zn sheets were placed in a transparent cuvette at a certain distance without contact, then electrolyte was added and connected to the LAND (CT3002A) battery testing system. The Zn plating behavior at a current density of 4 mA cm^-2^ was finally observed and recorded under an optical microscope.

**DFT Computations**

The density functional theory (DFT) calculations of precise structure optimization were performed using Gaussian 09. Geometry optimization and frequency calculations of all entities were carried out using the B3LYP functional and 6-31G* basis. The high-precision single-point energy was calculated at the PBE0-D37 /def2-TZVP6 theoretical level. The electrostatic potential (ESP) was calculated by the Multiwfn software.

The binding energy calculations were performed in the Vienna ab initio simulation packages (VASP),[1, 2] using the generalized gradient approximation (GGA) with Perdew–Burke–Ernzerhof (PBE) parameterization.[3-5] The Zn(002), Zn(100), Zn(101) surfaces were composed of 4 × 4, 4 × 2 and 2 × 4 supercells, respectively. A vacuum space of 20 Å was set to eliminate possible interaction between the periodic images. A 3 × 3 × 1 Monkhorst–Pack k-point mesh was used for the Brillouin zone integration. The energy cut-off of the plane wave basis was set as 450 eV. The convergence criteria of force and energy were set as 0.03 eVÅ^−1^ and 10^−5^ eV, respectively. The DFT-D3 method was applied to include the van der Waals interactions.[6] The adsorption energy is defined as:

ΔE_ads_ = ΔE_ads-sub_ − ΔE_i-ads_ − ΔE_sub_,

in which E_ads_-substrate, E_i-ads_, and E_sub_ are the energies of the adsorbate-substrate system, isolated adsorbates, and Zn substrate, respectively.

**Figure S1.** Optical photographs of different electrolytes at different temperatures.

**Figure S2.** Flammability of a) EF2 and b) ZEFW2 at 25 ℃.

**Figure S3.** TGA curves of the Zn(OAc)_2_ and ZEFW2 electrolytes showing their distinct temperature-dependent mass-loss behavior.

**Figure S4.** Nyquist plot of stainless steel||stainless steel cells with different electrolytes before cycling a) at 25 ℃ and b) at -20 ℃.

**Figure S5.** Ionic conductivities of different electrolytes a) at 25 ℃ and b) at -20 ℃.

**Figure S6.** CA curve at 10 mV and Nyquist plots of Zn||Zn cells with a) Zn(OAc)_2_ and b) ZEFW2 at 25 ℃.

**Figure S7.** CA curve at 10 mV and Nyquist plots of Zn||Zn cells with a) ZEFW1, b) ZEFW2 and c) ZEFW4 at -20 ℃.

**Figure S8.** Time-resolved a) radial distribution functions and b) cumulative coordination numbers for the dehydration of water molecules during the initial stage of ZEFW2 simulation.

**Figure S9.** Time-resolved a) radial distribution functions and b) cumulative coordination numbers for the coordination dynamics of formamide during the initial stage of ZEFW2 simulation.

**Figure S10.** a) and b) Nyquist plots of Zn||Zn cells with different electrolytes at 25 ℃. The inset shows the equivalent circuit for fitting Nyquist plots.

**Figure S11.** a) - c) Nyquist plots of Zn||Zn cells with different electrolytes at -20 ℃. The inset shows the equivalent circuit for fitting Nyquist plots.

**Figure S12.** a) AFM images and b) surface profile along the white line of Zn anodes after 100 cycles with different electrolytes at different temperatures.

**Figure S13.** SEM images and EDS mapping of the Zn metal surface in Zn||Zn cells after 20 cycles a) with Zn(OAc)_2_ and b) ZEFW2 at 25 ℃.

**Figure S14.** SEM images of the Zn metal surface in a Zn||Zn cell after 20 cycles with ZEFW2 at -20 ℃.

**Figure S15.** Fully optimized adsorption configurations on the Zn(002) surface. Fully optimized configurations of a) H_2_O, b) ethanol, c) FA, and d) EF adsorbed on Zn(002).

**Figure S16.** Fully optimized Zn^2+^ adsorption configurations on a) EF-modified Zn(002) and b) pristine Zn(002) surfaces.

**Figure S17.** Fully optimized adsorption configurations on the Zn(100) surface. Fully optimized configurations of a) H_2_O, b) ethanol, c) FA, and d) EF adsorbed on Zn(100).

**Figure S18.** Fully optimized Zn^2+^ adsorption configurations on a) EF-modified Zn(100) and b) pristine Zn(100) surfaces.

**Figure S19.** Fully optimized adsorption configurations on the Zn(101) surface. Fully optimized configurations of a) H_2_O, b) ethanol, c) FA, and d) EF adsorbed on Zn(101).

**Figure S20.** Fully optimized Zn^2+^ adsorption configurations on a) EF-modified Zn(101) and b) pristine Zn(101) surfaces.

**Figure S21.** XRD patterns of Zn anode after a) 1 cycle; b) 100 cycles at 25 ℃ with different electrolytes.

**Figure S22.** XRD patterns of Zn anode after different cycles in Zn||Zn cells with ZEFW2 at -20 ℃.

 **Figure S23.** a) and b) The fitted Ft-EXAFS spectra in R space.

**Figure S24.** a) - d) Wavelet transform images of the EXAFS spectra.

**Figure S25.** Nyquist plots of Zn||NVO cells with different electrolytes before cycling a) at 25 ℃ and b) at -20 ℃.

**Figure S26.** Nyquist plots of Zn||Zn cells with different electrolytes before cycling a) at 25 ℃ and b) at -20 ℃.

**Figure S27.** Tafel plots of Zn||Zn cells with different electrolytes before cycling at a) 25 ℃ and b) -20 ℃.

**Figure S28.** LSV response curves of different electrolytes measured in a Zn||Ti half-cell using linear potential scan at a scan rate of 1 mV s^-1^ before cycling a), b) at 25 ℃ and c), d) at -20 ℃.

**Figure S29.** CV profiles of Zn||stainless steel cells in different electrolytes at 25 ℃.

**Figure S30.** CA curves of the Zn anode tested in different electrolytes at an overpotential of -150 mV at 25 ℃.

**Figure S31.** CA curves of the Zn anode tested in ZEFW2 at an overpotential of -150 mV at different temperatures.

**Figure S32.** a) and b) Cycling performance of the Zn||Zn cells with different electrolytes at a current density of 0.5 mA cm^-2^ and an areal capacity of 0.25 mAh cm^-2^ at 25 ℃.

**Figure S33.** Rate performance and long-term performance of Zn||Zn cells with Zn(OAc)_2_ and ZEFW2 at 25 ℃.

**Figure S34.** Cycling performance of the Zn||Zn cell with ZEFW2 at a current density of 0.5 mA cm^-2^ and an areal capacity of 0.5 mAh cm^-2^ at -20 ℃.

**Figure S35.** Rate performance and long-term performance of Zn||Zn cell with ZEFW2 at -20 ℃.

**Figure S36.** CV curves of Zn||NVO cell operated at different cycles with Zn(OAc)_2_ at 1 mV s^-1^ at 25℃.

**Figure S37.** CV curves of Zn||NVO cells operated with Zn(OAc)_2_ and ZEFW2 at at 1 mV s^-1^ 25 ℃.

**Figure S38.** Rate performance of Zn||NVO cells operated with Zn(OAc)_2_ and ZEFW2 at 25 ℃.

**Figure S39.** Rate performance of Zn||NVO cells with ZEFW2 at -20 ℃.

**Figure S40.** the lighted LEDs by pouch cell.

**Figure S41.** Long term cycling performance of Zn||NVO cells at 1 A g^-1^ at 25 ℃.

**Figure S42.** Long term cycling performance of Zn||NVO pouch cell at 0.5 A g^-1^ at 25 ℃.

**Figure S43.** a) Photograph of the ZEFW2-based pouch cell during electrochemical testing. b) Photograph showing the dimensions of the pouch cell.

**Figure S44.** a) Voltage-specific capacity profiles of the ZEFW2-based pouch cell at -20°C at selected cycle numbers. b) Discharge capacity of the ZEFW2-based pouch cell as a function of cycle number at -20 °C.

**Figure S45.** a) Voltage-specific capacity profiles of the ZEFW2-based pouch cell at 25 °C at selected cycle numbers. b) Voltage-specific capacity profiles of the Zn(OAc)_2_-based pouch cell at 25 °C at selected cycle numbers. c) Discharge capacity of ZEFW2- and Zn(OAc)_2_-based pouch cells as a function of cycle number at 25 °C.

**Table S1.** Peak-fitting parameters of the O-H stretching band for Zn(OAc)_2_ and ZEFW2 electrolytes.

| Electrolyte | Temperature | Component | FWHM (cm^-1^) | Area | Area ratio (%) |
| --- | --- | --- | --- | --- | --- |
| Zn(OAc)_2_ | RT | SHW | 175.10 | 5922.37 | 27.42 |
|  |  | WHW | 248.64 | 15025.71 | 69.57 |
|  |  | NHW | 88.08 | 649.19 | 3.01 |
| ZEFWE2 | RT | SHW | 136.31 | 2939.73 | 19.2 |
|  |  | WHW | 260.14 | 12187.32 | 79.60 |
|  |  | NHW | 58.01 | 184.20 | 1.2 |
| ZEFWE2 | -20°C | SHW | 132.73 | 2929.0 | 20.4 |
|  |  | WHW | 242.64 | 11164.80 | 77.76 |
|  |  | NHW | 71.26 | 263.96 | 1.84 |

**Table S2**. Depth-resolved elemental composition of the ZEFW2-derived Zn interphase determined by XPS.

| **Sputtering depth (nm)** | **O 1s (Atomic %)** | **Zn 2p (Atomic %)** | **C 1s (Atomic %)** | **N 1s (Atomic %)** |
| --- | --- | --- | --- | --- |
| 0 | 46.6947 | 30.3674 | 21.5959 | 1.34192 |
| 10 | 48.4496 | 34.1752 | 16.1831 | 1.19203 |
| 20 | 48.2238 | 34.9734 | 14.9591 | 1.84371 |
| 30 | 49.2475 | 36.8073 | 12.3887 | 1.5564 |
| 40 | 48.6399 | 37.609 | 12.1054 | 1.64565 |
| 50 | 48.5725 | 38.4395 | 11.4393 | 1.54872 |

**Table S3**. Depth-resolved elemental composition of the Zn(OAc)_2_-derived Zn interphase determined by XPS.

| **Sputtering depth (nm)** | **O 1s (Atomic %)** | **Zn 2p (Atomic %)** | **C 1s (Atomic %)** |
| --- | --- | --- | --- |
| 0 | 41.0431 | 20.6630 | 38.2939 |
| 10 | 43.7498 | 36.1410 | 20.1093 |
| 20 | 44.6186 | 39.8260 | 15.5554 |
| 30 | 44.3660 | 42.1793 | 13.4547 |
| 40 | 43.8356 | 43.2691 | 12.8954 |
| 50 | 43.0003 | 45.4268 | 11.5729 |

**Table S4.** Calculated adsorption energies of the optimized configurations.

| **Zn surface** | **Adsorbate / configuration** | **Adsorption energy, Eads (eV)** |
| --- | --- | --- |
| Zn(002) | H_2_O-Zn(002) | -0.2662 |
| Zn(002) | Ethanol-Zn(002) | -0.5082 |
| Zn(002) | FA-Zn(002) | -0.5245 |
| Zn(002) | EF-Zn(002) | -0.8839 |
| Zn(002) | EF-modified Zn(002) + Zn^2+^ | 0.9145 |
| Zn(002) | Pristine Zn(002) + Zn^2+^ | 1.0606 |
| Zn(100) | H_2_O-Zn(100) | -0.4478 |
| Zn(100) | Ethanol-Zn(100) | -0.6292 |
| Zn(100) | FA-Zn(100) | -0.4471 |
| Zn(100) | EF-Zn(100) | -0.8969 |
| Zn(100) | EF-modified Zn(100) + Zn^2+^ | 0.8965 |
| Zn(100) | Pristine Zn(100) + Zn^2+^ | 0.9424 |
| Zn(101) | H_2_O-Zn(101) | -0.5409 |
| Zn(101) | Ethanol-Zn(101) | -0.7666 |
| Zn(101) | FA-Zn(101) | -0.7657 |
| Zn(101) | EF-Zn(101) | -1.1724 |
| Zn(101) | EF-modified Zn(101) + Zn^2+^ | 0.2227 |
| Zn(101) | Pristine Zn(101) + Zn^2+^ | 0.3515 |

**Table S5.** Curvefit Parameters for Zn K-edge EXAFS of ZEFW2.

| Path | e0 | N | R/ Å | σ^2^/10^-3^Å^2^ | R factor |
| --- | --- | --- | --- | --- | --- |
| Zn-O | 6.81 ± 3.23 | 3.21 ± 1.15 | 1.91 ± 0.06 | 3.90 ± 1.34 | 1.87% |
| Zn-N |  | 2.82 ± 0.98 | 1.94 ± 0.03 | 8.97 ± 2.17 |  |
| Zn-Zn |  | 6.50 ± 1.22 | 2.83 ± 0.04 | 9.37 ± 3.75 |  |
| Zn-C |  | 1.01 ± 0.19 | 3.54 ± 0.12 | 5.48 ± 1.57 |  |

S_0_^2^ was fixed as 1.0. Data ranges: 2.0 ≤ k ≤11.0 Å^-1^, 1.0 ≤ R ≤ 4.0 Å. The distances for Zn-O was from the FEFF crystal structure of ZnO, Zn-Zn was from the FEFF crystal structure of Zn, Zn-N and Zn-C were from the FEFF crystal structure of Zn-Phthalocyanine.

**Table S6.** Curvefit Parameters for Zn K-edge EXAFS of ZEFW2-50 cycles.

| Path | e0 | N | R/ Å | σ^2^/10^-3^Å^2^ | R factor |
| --- | --- | --- | --- | --- | --- |
| Zn-O | 9.28 + 0.14 | 4.10 ± 1.12 | 2.02 ± 0.05 | 9.41 ± 3.56 | 1.75% |
| Zn-N |  | 2.01 ± 0.32 | 2.05 ± 0.06 | 4.48 ± 1.18 |  |
| Zn-Zn |  | 8.07 ± 1.89 | 2.90 ± 0.03 | 2.71 ± 0.85 |  |
| Zn-C |  | 1.22 ± 0.13 | 3.32 ± 0.10 | 6.99 ± 1.63 |  |

S_0_^2^ was fixed as 1.0. Data ranges: 2.0 ≤ k ≤11.0 Å^-1^, 1.0 ≤ R ≤ 4.0 Å. The distances for Zn-O was from the FEFF crystal structure of ZnO, Zn-Zn was from the FEFF crystal structure of Zn, Zn-N and Zn-C were from the FEFF crystal structure of Zn-Phthalocyanine.

**Table S7.** Curvefit Parameters for Zn K-edge EXAFS of Zn(OAc)_2_.

| Path | e0 | N | R/ Å | σ^2^/10^-3^Å^2^ | R factor |
| --- | --- | --- | --- | --- | --- |
| Zn-O | 8.30 + 2.67 | 3.81 ± 0.87 | 2.00 ± 0.03 | 6.22 ± 2.28 | 2.69% |
| Zn-O (Zn_5_(OH)_6_(CO_3_)_2_) |  | 9.51 ± 2.29 | 2.21 ± 0.02 | 7.26 ± 3.01 |  |
| Zn-Zn |  | 1.52 ± 0.24 | 2.66 ± 0.05 | 2.53 ± 0.94 |  |

S_0_^2^ was fixed as 1.0. Data ranges: 2.0 ≤ k ≤11.0 Å^-1^, 1.0 ≤ R ≤ 3.0 Å. The distances for Zn-O was from the FEFF crystal structure of ZnO, Zn-Zn was from the FEFF crystal structure of Zn, second Zn-O were from the FEFF crystal structure of Zn_5_(OH)_6_(CO_3_)_2_.

**Table S8.** Curvefit Parameters for Zn K-edge EXAFS of Zn(OAc)_2_-cycles.

| Path | e0 | N | R/ Å | σ^2^/10^-3^Å^2^ | R factor |
| --- | --- | --- | --- | --- | --- |
| Zn-O | 6.35 + 2.08 | 4.50 ± 1.17 | 2.01 ± 0.04 | 1.74 ± 0.68 | 1.73% |
| Zn-O (Zn_5_(OH)_6_(CO_3_)_2_) |  | 2.88 ± 0.33 | 2.20 ± 0.01 | 6.85 ± 3.40 |  |
| Zn-Zn |  | 7.07 ± 1.76 | 2.65 ± 0.04 | 5.76 ± 2.87 |  |

S_0_^2^ was fixed as 1.0. Data ranges: 2.0 ≤ k ≤11.0 Å^-1^, 1.0 ≤ R ≤ 3.0 Å. The distances for Zn-O was from the FEFF crystal structure of ZnO, Zn-Zn was from the FEFF crystal structure of Zn, second Zn-O were from the FEFF crystal structure of Zn_5_(OH)_6_(CO_3_)_2_.

**Table S9.** Literature data used for the multidimensional comparison of cycling performance at -20 ℃.

| **Electrolyte** | **Time (h)** | **Areal capacity (mAh cm^-2^)** | **Current density (mA cm^-2^)** | **Temperature (℃)** | **Reference** |
| --- | --- | --- | --- | --- | --- |
| **ZEFW2** | 1400 | 10 | 1 | -20 | **This work** |
| **ZEFW2** | 2000 | 0.5 | 0.5 | -20 | **This work** |
| **EG/ZS** | 80 | 1 | 2 | -20 | [7] |
| **EmimAc/PVA** | 1300 | 0.1 | 0.1 | -20 | [8] |
| **PVA/DMSO/Agar** | 500 | 0.2 | 0.2 | -20 | [9] |
| **PVA/ZDES** | 600 | 0.5 | 0.5 | -20 | [10] |
| **GG/SA/EG** | 200 | 0.2 | 0.2 | -20 | [11] |
| **PVA/KAc** | 1200 | 0.1 | 0.5 | -20 | [12] |
| **APM** | 2000 | 0.25 | 0.5 | -20 | [13] |
| **SA-TreH** | 200 | 0.25 | 0.5 | -20 | [14] |
| **PAM/ZS/GL/AN** | 500 | 0.2 | 0.2 | -20 | [15] |
| **ZS/LiTSI** | 500 | 0.2 | 0.2 | -20 | [16] |

**Table S10.** Quantitative summary of pouch-cell parameters and electrochemical performance.

| **Parameter** | **ZEFW2** | **Zn(OAc)_2_** |
| --- | --- | --- |
| Dimension | 5*4.5 cm^2^ | 5*4.5 cm^2^ |
| Active material loading@25 ℃ | 0.974 mg cm^-2^ | 0.930 mg cm^-2^ |
| 25 ℃, cycle 1 | 0.00643 Ah | 0.00513 Ah |
| 25 ℃, cycle 100 | 0.00219 Ah | 0.00143 Ah |
| 25 ℃, cycle 500 | 0.00112 Ah | 0.00036 Ah |
| -20 ℃ operation | Yes | No stable operation |
| Active material loading@-20 ℃ | 1.412 mg cm^-2^ | - |
| -20 ℃, cycle 1 | 0.00447 Ah | - |
| -20 ℃, cycle 100 | 0.00432 Ah | - |
| -20 ℃, cycle 406 | 0.00418 Ah | - |

**Reference**

[1] G. Kresse, J. Furthmuller, "Efficient iterative schemes for ab initio total-energy calculations using a plane-wave basis set," Phys Rev B Condens Matter **54** (1996): 11169-11186.

[2] G. Kresse, J. Furthmüller, "Efficiency of ab-initio total energy calculations for metals and semiconductors using a plane-wave basis set," Computational Materials Science **6** (1996): 15-50.

[3] J.P. Perdew, K. Burke, M. Ernzerhof, "Generalized Gradient Approximation Made Simple," Physical Review Letters **77** (1996): 3865-3868.

[4] P.E. Blochl, "Projector augmented-wave method," Phys Rev B Condens Matter **50** (1994): 17953-17979.

[5] G. Kresse, D. Joubert, "From ultrasoft pseudopotentials to the projector augmented-wave method," Physical Review B **59** (1999): 1758-1775.

[6] S. Grimme, J. Antony, S. Ehrlich, H. Krieg, "A consistent and accurate ab initio parametrization of density functional dispersion correction (DFT-D) for the 94 elements H-Pu," J Chem Phys **132** (2010): 154104.

[7] N. Chang, T. Li, R. Li, S. Wang, Y. Yin, H. Zhang, X. Li, "An aqueous hybrid electrolyte for low-temperature zinc-based energy storage devices," Energy & Environmental Science **13** (2020): 3527-3535.

[8] Y. Chen, M. Zhu, C. Li, H. Wang, D. Chen, H. Wu, Z. Huang, Y. Wang, Y. Fan, Z. Bai, "onic liquid‐based hydrogel electrolytes enabling high‐voltage‐plateau zinc‐ion batteries," Advanced Functional Materials **35** (2025): 2501162.

[9] M. Li, C. Xi, X. Wang, L. Li, Y. Xiao, Y. Chao, X. Zheng, Z. Liu, Y. Yu, C. Yang, Spontaneous desaturation of the solvation sheath for high‐performance anti‐freezing zinc‐ion gel‐electrolyte, Small **19** (2023): 2301569.

[10] Q. Peng, R. Cao, H. Ma, Y. Li, E. Zhu, X. Zhu, N. Ma, M. Xue, X. Zhang, "One-step preparation of an anti-freezing and flame-retardant eutectic gel electrolyte for assembling a flexible and high-performance zinc-ion battery," Journal of Materials Chemistry A **13** (2025): 6586-6596.

[11] J. Wang, Y. Huang, B. Liu, Z. Li, J. Zhang, G. Yang, P. Hiralal, S. Jin, H. Zhou, "Flexible and anti-freezing zinc-ion batteries using a guar-gum/sodium-alginate/ethylene-glycol hydrogel electrolyte," Energy Storage Materials **41** (2021): 599-605.

[12] Y. Yan, S. Duan, B. Liu, S. Wu, Y. Alsaid, B. Yao, S. Nandi, Y. Du, T.W. Wang, Y. Li, "Tough hydrogel electrolytes for anti‐freezing zinc‐ion batteries," Advanced Materials **35** (2023): 2211673.

[13] Y. Liu, L. Miao, H. Shen, Z. Wang, K. Yao, Y. Hu, J. Sun, S. Hou, J. Zhao, K. Yang, "Maximizing functional diversity of electrolyte additives through modular molecular engineering to stabilize zinc metal anodes," Advanced Functional Materials **35** (2025): 2501968.

[14] Q. Bai, Q. Meng, W. Liu, W. Lin, P. Yi, J. Tang, G. Zhang, P. Cao, J. Yang, "Advanced electrolyte with high stability and low-temperature resistance for zinc-ion batteries," Journal of Materials Chemistry A **12** (2023): 277-285.

[15] T. Wei, Y. Ren, Z. Li, X. Zhang, D. Ji, L. Hu, "Bonding interaction regulation in hydrogel electrolyte enable dendrite-free aqueous zinc-ion batteries from -20 to 60° C," Chemical Engineering Journal **434** (2022): 134646.

[16] J. Wu, M. Li, X. Ding, Z. Chen, J. Luo, Q. Zhang, Y. Qiu, Q. Wang, W. Liu, C. Yang, "Upgrading Gel Electrolytes Through Electrostatic‐Induced Dual‐Salt Paradigm for Superior Zn‐Ion Battery Performance," Small **20** (2024): 2400390.
